# Online Learning for Surgical Skill in Clinical-year Medical Students: a Rapid Review

**DOI:** 10.1007/s40670-024-02093-x

**Published:** 2024-06-08

**Authors:** Azizi Sheik-Ali

**Affiliations:** University of Exeter Medical School, 79 Heavitree Rd, EX2 4TH, Exeter, England

**Keywords:** Online learning, Curriculum design, Surgical skills, Surgical education

## Abstract

**Background:**

Surgical skills are essential competencies in medical education. All doctors registered with the General Medical Council (GMC) are required to perform surgical tasks safely and effectively. There are no reviews specifically investigating the use of online learning in teaching surgical skills for medical students in their clinical years. The aim of this rapid review was to investigate the effectiveness and perceptions of online teaching of surgical skills for medical students in their clinical years.

**Methods:**

A rapid review was performed of the MEDLINE and EMBASE database in line with Preferred Reporting Items for Systematic Reviews and Meta-Analyses guidelines. The quality of the searched articles was evaluated using the Medical Education Research Study Quality Instrument. Both observational studies and randomised controlled trials were included in the review if they met the inclusion criteria of involving medical students in their clinical years, online learning, and surgical-related skills or content.

**Findings:**

Our search strategy yielded 140 studies. Eleven studies were included in the review equating to an analysis of 636 medical students. The results indicate that online teaching of surgical skills allows improved surgical skill acquisition, with medical students having an overall positive perception towards it.

**Conclusion:**

This rapid review suggests that online teaching of surgical skills can be effective for medical students in their clinical years in surgical skill acquisition and positive medical student perception. Further studies with larger sample sizes are required to support the conclusions of this review.

## Background

The General Medical Council (GMC) requires all doctors registered with the GMC to be able to perform surgically related tasks safely and effectively [1]. These include the suturing, handling of surgical instruments, and knot tying. There is a clear need for medical schools to incorporate this teaching in the medical curriculum.

Surgical skills are primarily taught through in-person tutorials by a trained physician and practised in both hospital attachments and skill laboratories. Most institutions incorporate surgical skill tutorials during the traditionally termed ‘clinical years’, which is usually from the third year of education onwards [2–4]. However, following the COVID-19 pandemic, a period emerged where social distancing measures led to the replacement of face-to-face hospital attachments and surgical skill tutorials with online teaching [5]. Online learning had already been seen as an important part of the future development of medical education [6, 7]. The COVID-19 pandemic accelerated this process [8].

Post COVID-19 pandemic, many medical institutions continue to opt to integrate online learning in their curriculums [9]. Reasons cited included reduced costs and more widely accessible learning not limited to geography [6]. There are no current reviews specifically investigating the use of online learning in teaching surgical skills for medical students in their clinical years. This review aims to investigate the effectiveness of online teaching of surgical skills, and the perception of medical students in their clinical years towards it.

## Method

A rapid review was conducted in line with the Preferred Reporting Items for Systematic Reviews and Meta-Analyses (PRISMA) guidelines to ensure methodological rigour [10]. A literature search of the MEDLINE and EMBASE databases was performed. Records from inception until November 2022 were reviewed. Search terms included ‘medical student’, ‘online learning’, and ‘surgical skills’. Qualitative analysis was performed on both observational studies and randomised controlled trials.

No exclusions were made based on publication date. The Medical Education Research Study Quality Instrument (MERSQI) was used to evaluate the quality of the searched articles. The search method is depicted as a modified PRISMA flowchart (Appendix Fig. 1).

### Inclusion Criteria

Studies were included if the following criteria were met:

(1) online learning; (2) medical students in their clinical years of practise/clinical rotations; (3) surgical-related skills or content.

### Exclusion Criteria

Articles involving medical students not in their clinical years of practise. In addition, studies teaching medical students’ procedures that utilised methods other than video-based learning and non-related surgical content.

## Findings

Our search strategy yielded 104 studies. After screening, 11 studies were included in this review. The studies were of heterogenous quality (MERSQI range 9.6–15). Among the 11 studies, there were a total of 636 medical students. All were in their clinical years (third year or above or explicitly stated). Participants attended a surgical curriculum comprising of online courses or combined online and face-to-face teaching. The surgical skills taught included suturing, knot tying, and instrument identification.

### Methods of Surgical Education

Interventional methods for online education included the following:

- Live web-based video [11, 14–17, 20].
- Self-directed video [12, 13, 18, 21].
- Both web-based video and standard conventional teaching [19].

The primary outcomes for all studies included were post-intervention proficiency, satisfaction or confidence in surgically related skills, and knowledge. Five studies used the objective structured assessment of technical skills (OSATS) to measure proficiency [11, 13, 18, 20, 21]. The results of each study are summarised as a table (Appendix Table 1).

### Confidence and Satisfaction of Participants Using Online Learning

Six studies assessed the satisfaction or confidence of online learning by medical students.

Through online teaching, Quaranto et al. [17] found a significant increase in the confidence of medical students in both knot tying (*p* = 0.03) and suturing (*p* < 0.001) pre and post programme.

Similarly, Shin et al. [15] found that following a virtual case-based curriculum, students had significantly improved self-confidence to independently complete a surgical clerkship by the end of the programme (5-point Likert scale: pre-programme 2.0 and post-programme 4.0, *p* = 0.0001). Here, students were presented with a surgically related case and students would take a medical history, request physical examinations, and discuss differential diagnoses.

Newcomb et al. found. [14] students reported increased confidence in gaining trust and building rapport of surgical patients through online video communication. The virtual class utilised role playing and feedback from simulated patients. Here, students explored understanding, concerns, and perception of patients as well as forming action treatment plans. Eighty percent of students rated this curriculum ‘A + ’.

McGann et al. [13] implemented online programmes where suturing, surgical instrument identification, and knot tying were used to teach surgical skills. They found a significant increase in confidence of students regarding suturing, surgical instrument identification, and knot tying (*p* < 0.0001, *p* < 0.0001, *p* < 0.0001, respectively). Students also felt positively regarding learning and interacting with the instructor through online platforms.

Bochenska et al. [19] recorded high student satisfaction in both video and non-video groups and de Sena et al.  [20] reported all participants found video-based learning was more favourable compared to text-based learning.

### Proficiency of Surgical Skills and Knowledge Through Online Learning

For skill competency, four comparative studies found no significant difference between the control and video-based learning group (face-to-face) for the education of procedural surgical skills [11, 18, 19, 21]. Two of these studies measured skill capability at longer periods of time and reported no difference between the video-based learning groups and control. [11, 19] Two other studies highlighted the improvement in surgical knowledge with the addition of video-based learning as an adjunct [12, 25]. 

Co et al. [11] utilised web-based learning of surgical skills. Here, demonstrations by tutors and practice by students of surgical skills were conducted on online platforms. They reported no significant difference between the face-to-face and web-based learning groups following OSATS (4.8/5 and 4.7/5, respectively, *p* = 1.0).

Shin et al. [15] found a significant improvement in assessment score for knowledge regarding surgically related content through a virtual case-based curriculum from pre and post programme (*p* value 0.0002). Similarly, Pettitt-Schieber et al. [21] recorded a significant increase in knowledge of the surgical subspecialty programme after education through online platforms (*p* < 0.0001).

Handaya et al. [12] reported that students that received a video for knot tying scored significantly higher compared to those that did not watch the video (*p* < 0.0001). Following assessment by OSATS, de Sena et al. [20] recorded a better performance in those receiving online education compared to text-based education (post-test results (*p* < 0.001).

## Discussion

Universities are increasingly discovering methods to incorporate online learning into their medical curriculum [9]. The findings from this review article indicate that there is a growing body of research on the effectiveness of online learning for surgically related skills, and that online learning can be an effective tool for enhancing the surgical skills of medical students either as a replacement or an adjunct to conventional methods [11, 18, 19, 21–23].

All studies highlighted effectiveness of online learning in improving medical students’ knowledge base and surgical skill proficiency. Categorising the results showed the following improvements: 1, Knowledge/Skill development; 2, Student satisfaction.

1. Improvements in knowledge base: 6 articles investigating pre- and post-acquisition of surgical skill theoretical knowledge and skill showed improvements in students recall post virtual teaching [11, 12, 15, 18, 19, 21]. A possible reason for this is the repeated accessibility to material and individualised learning pace. Unlike in a traditional classroom setting, learners can pause and rewind videos, practice a technique, or move on to the next lesson when they feel ready [24]. This can be helpful for learners who require individualised learning plans. This is consistent with the current literature on the general understanding that online learning can increase student engagement [22, 25].
2. Improvements in student satisfaction: Six articles highlighted moderate or major improvements in student confidence and/or satisfaction in performing surgical skills with video-based learning [13–15, 17, 19, 20]. Online learning for surgical skills provides access to an array of training materials and resources. Further, faculty are not limited to the location of the medical institution; therefore, the pool of expert knowledge to provide students guidance increases [6].

However, some limitations regarding online learning exist, such as deteriorating mental health, social isolation, and poor concentration [26, 27]. Nishimura et al. [26] found worsening mental health among medical students when shifting to online learning. This may be due to prolonged periods of isolation. Similarly, Ray et al. [27] found that student concentration was poor due to distractions during an online course. A limitation here was the use of subjective questionnaires and no direct comparison to traditional methods.

Each method of online teaching offers unique advantages. Live web-based video instruction shows promise in enhancing student engagement. It provides a clear platform for demonstrating practical skills, ensuring equal visibility for all students [11].

Furthermore, it eliminates the necessity for physical presence on campus, thereby increasing accessibility for students. Previous research corroborates these benefits of online learning [28, 29].

Self-directed video learning provides learners with flexibility and autonomy, in accordance with learner-centred pedagogy principles [30]. Learners can replay lectures at their convenience. Additionally, students can control the pace of the lecture, facilitating a personalised learning experience. This flexibility accommodates varied learning styles.

Research has indicated that integrating web-based videos into medical student curricula enhances learning outcomes [19]. For instance, providing access to knot tying boards for suturing outside of the clinical environment facilitated additional practice opportunities, augmenting skill development beyond traditional settings. This integration extends learning beyond the confines of the classroom, promoting continuous improvement.

The current evidence suggests that online learning can be an effective tool for enhancing surgically related skills of clinical medical students. The current literature outside of surgically acquired skills states that online learning can be an effective adjunct to traditional teaching [31]. It may be wise to presume this is the case too for surgical skill acquisition until more in-depth studies with larger sample sizes are completed. We suggest the effectiveness of the mode of delivery in online education varies depending on the type of content being taught. For instance, practical skills may benefit more from a combination of live web-based instruction and self-directed video learning, ensuring both engagement and flexibility in skill acquisition.

This review has many limitations due to the heterogeneity of the primary studies, including differences in explored primary outcomes. One major critique includes the current limited number of studies available evaluating clinical medical students and e-learning in surgery. It is therefore difficult to draw direct conclusions from this review based on the small sample size; however, it provides a starting point for further research. Although four studies explored medical students’ surgical skill proficiency and compared it to traditional methods, the sample sizes were small, and the skill sets of the students prior to teaching were not clearly established (Appendix Table 1). Furthermore, there was potential selection bias in the Shin et al. study. It appears the authors contributed to the formation of the pre- and post-intervention questionnaire and selection of students. A blind selection process of students, including records of the students’ baseline skill sets and clarity on the completion date for feedback questionnaires post-intervention, would improve the study reliability.

The heterogeneity in primary outcomes and statistics makes it difficult to combine the results; however, there was a clear trend in improvements in confidence levels of medical students post online intervention and knowledge base in the studies examined.

## Conclusion

In conclusion, this review suggests online teaching of surgical skills may be effective for medical students in their clinical years in acquisition of surgical skill. The review also found that medical students positively perceive online learning of surgical skills. Further studies are needed to fully understand the effectiveness of online learning for surgical skills, either as an adjunct or as a replacement of traditional methods, and to determine the best way to incorporate it into medical education curriculums.

## Figures and Tables

**Fig. 1:**
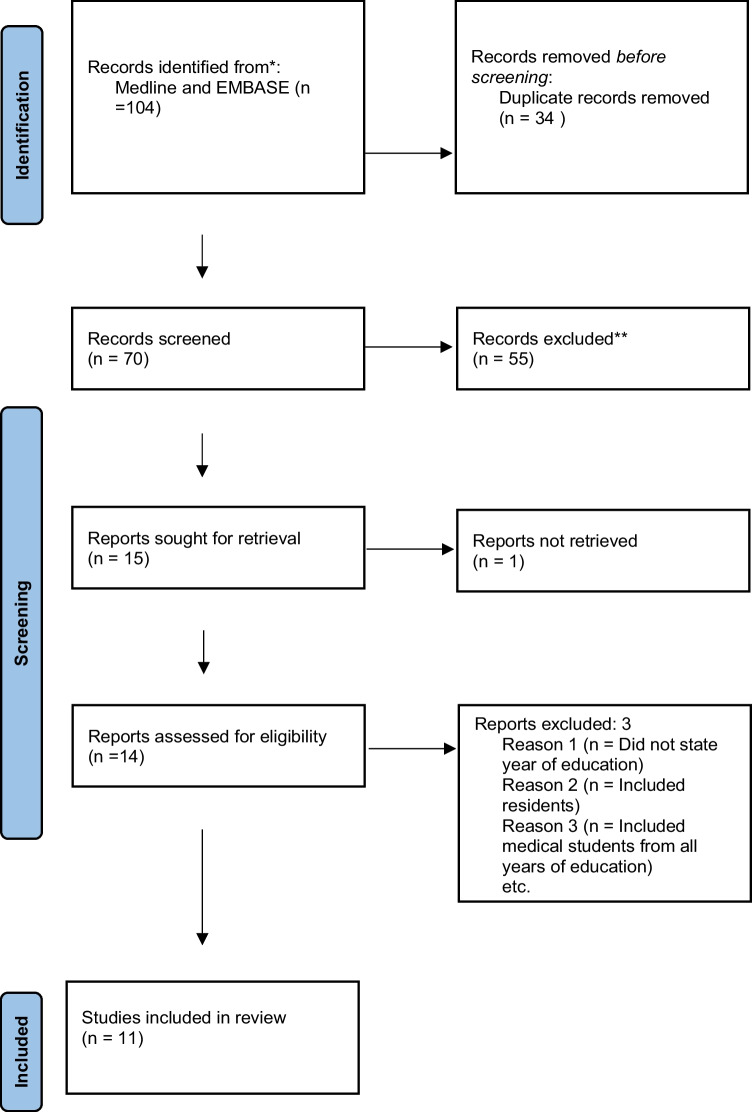
The modified PRISMA flowchart for search method

## Data Availability

Not applicable.

## Appendix

Figure 1

Table 1

**Table 1:** Summary of studies with design, population, intervention, findings, and MERSQI scores

Author, year	Study design and method	Population	Curriculum type	Key findings	MERSQI
Co et al., 2021 [11]	Prospective case–control study	62 final year students	Online demonstration and real-time practice for: incision, suturing, and knot tying Compared with conventional face-to-face teaching of skills	No significant difference between objective structured assessment of technical skills (OSATS) intervention and control group	13.8
Handaya et al., 2021 [12]	Prospective cohort study	89 medical students on surgical clinical rotation	Tutorial video combined with online class on knot tying. Video shown either ‘pre-online class’ or ‘post-online class’ Compared to group with no tutorial video	Pre-online class: higher scores for knot tying (*p* = 0.000*) Post-online class: no significant difference between the two groups	13.8
McGann et al., 2021 [13]	Single cohort pre- and post-test	86 3rd and 4th year medical students	Online 1-week elective, on surgical skills: knot tying and suturing	Nearly all students met course objectives and reported significant increase in confidence Positive objective student performance across all areas of surgical teaching	10.8
Newcomb et al., 2021 [14]	Single cohort pre- and post-test	5 4th year medical students	Virtual class with role play and group debrief	Improved student overall confidence and rapport building skills in video conference communication Positive qualitative feedback	9.6
Shin et al., 2021 [15]	Single cohort pre- and post-test	16 3rd year medical students	Virtual surgery clerkship curriculum including case-based learning and role play on key surgical specialty topics	All students agreed that the course met all their learning objectives. There was an increase in clinical confidence after the completion of the virtual curriculum (*p* = 0.0001). The course improved knowledge retention (*p* = 0.0002) and increased career interest	13.2
Pettitt et al., 2021 [16]	Single cohort pre- and post-test	67 3rd and 4th year medical students	Virtual surgical electives covering lectures, conferences, and surgical skills (suturing knot tying)	Improvement in post-test scores across all specialties after the virtual surgical electives. On average, all speciality courses achieved statistically significant increase in self-reported student knowledge and understanding (*p* = 0.021– < 0.0001)	10.8
Quaranto et al., 2021 [17]	Single cohort pre- and post-test	31 3rd year medical students	Remote interactive surgical skills sessions covering instrument identification, knot tying, and suturing	Significant increase in confidence score on knot tying (7.9 ± 0.7 ➔ 9.7 ± 0.9, *p* = 0.03) and suturing (8.0 ± 1.3 ➔ 13.8 ± 0.9, *p* < 0.001)	10.8
Lwin et al., 2018 [18]	Randomised controlled trial	50 final year medical students	Self-directed interactive video-based learning covering basic surgical skills (suturing, knot tying) compared with in-person instructor led teaching	Mean OSATS scores increased after the intervention (*p* < 0.001) There was not a significant difference in post-intervention scores between the video-based learning compared to instructor led teaching	14.4
Bochenska et al., 2018 [19]	Prospective randomised controlled trial	50 3rd year medical students	Web-based expert knot tying video compared to standard in person clinical skills curriculum	There was a significant increase in student performance on knot tying for both groups from pre- and post-intervention (EV: *p* = 0.004, SC: *p* < 0.001)	15
De sena et al., 2013 [20]	Randomised controlled trial	50 5th and 6th year medical students	Computer-assisted learning guide compared to printed text ‘how to’ guide on rhomboid flap technique	The computer-assisted learning group had significantly better performance when compared to the text-based education group (*p* < 0.002), overall global assessment (*p* = 0.017), and post-test results (*p* < 0.001)	15
Tejos et al., 2020 [21]	Prospective randomised controlled trial	130 5th year medical students	Comparison of video-guided learning, peer feedback and expert feedback for teaching suturing skills	Post-assessment results of the peer-feedback and expert feedback groups were significantly superior to the video-guided learning group in OSATS scores (*p* < 0.05). Video-guided learning alone is not enough	15
